# Use of a Connected Glucose Meter and Certified Diabetes Educator Coaching to Decrease the Likelihood of Abnormal Blood Glucose Excursions: The Livongo for Diabetes Program

**DOI:** 10.2196/jmir.6659

**Published:** 2017-07-11

**Authors:** Janelle Downing, Jenna Bollyky, Jennifer Schneider

**Affiliations:** ^1^ Center of Health and Community University of California, San Francisco San Francisco, CA United States; ^2^ Livongo Health Mountain View, CA United States; ^3^ Department of Medicine Stanford University Medical Center Stanford, CA United States

**Keywords:** SMBG, blood glucose self-monitoring, diabetes remote monitoring, diabetes management, diabetes mellitus, self-care

## Abstract

**Background:**

The Livongo for Diabetes Program offers members (1) a cellular technology-enabled, two-way messaging device that measures blood glucose (BG), centrally stores the glucose data, and delivers messages back to the individual in real time; (2) unlimited BG test strips; and (3) access to a diabetes coaching team for questions, goal setting, and automated support for abnormal glucose excursions. The program is sponsored by at-risk self-insured employers, health plans and provider organizations where it is free to members with diabetes or it is available directly to the person with diabetes where they cover the cost.

**Objective:**

The objective of our study was to evaluate BG data from 4544 individuals with diabetes who were enrolled in the Livongo program from October 2014 through December 2015.

**Methods:**

Members used the Livongo glucose meter to measure their BG levels an average of 1.8 times per day. We estimated the probability of having a day with a BG reading outside of the normal range (70-180 mg/dL, or 3.9-10.0 mmol/L) in months 2 to 12 compared with month 1 of the program, using individual fixed effects to control for individual characteristics.

**Results:**

Livongo members experienced an average 18.4% decrease in the likelihood of having a day with hypoglycemia (BG <70 mg/dL) and an average 16.4% decrease in hyperglycemia (BG >180 mg/dL) in months 2-12 compared with month 1 as the baseline. The biggest impact was seen on hyperglycemia for nonusers of insulin. We do not know all of the contributing factors such as medication or other treatment changes during the study period.

**Conclusions:**

These findings suggest that access to a connected glucose meter and certified diabetes educator coaching is associated with a decrease in the likelihood of abnormal glucose excursions, which can lead to diabetes-related health care savings.

## Introduction

Diabetes mellitus affects over 387 million people worldwide. Its prevalence has increased dramatically over the past two decades (from 9.8% to 12.4% in the United States), with the costs of diabetes now estimated at over US $150 billion annually in the United States [1-3]. Blood glucose (BG) excursions out of the normal range (70-180 mg/dL, or 3.9-10.0 mmol/L [4]) are important drivers of these extraordinary costs, often leading to unnecessary emergency department visits, hospitalization, urgent care visits, and office visits as well as missed work days.

Self-monitoring of BG (SMBG) is an integral part of successful diabetes management. Self-monitoring of BG has been demonstrated to be a beneficial approach for the achievement of long-term glycemic control in patients with both type 1 [5] and type 2 diabetes [6]. It also supports preventive strategies for acute and chronic complications of diabetes by increasing a patient’s awareness of hyperglycemia and hypoglycemia [7].

Traditional BG meters provide glucose measurements and store these data within the meter. These data must be actively retrieved and analyzed by the user, health care provider, software, or mobile app to guide treatment decisions [3,8-10]. The Livongo for Diabetes Program, currently available in the United States which is offered directly to patients or as a benefit through their self-insured employer, provider, or health insurance, leverages cloud technology to offer patients (1) a cellular technology-enabled, two-way messaging device that measures BG, centrally stores the glucose data and other contextual data, and delivers relevant algorithmic messages back to the individual; (2) unlimited glucose test strips, and (3) access to a team of certified diabetes educators (CDEs) for questions, goal setting, and immediate support in the setting of extreme glucose excursions.

An “alert” interaction between patients and a Livongo CDE coach occurs when a BG value transmitted through the meter is over 400 mg/dL (22.2 mmol/L) or below 50 mg/dL (2.8 mmol/L), or whatever thresholds a member elects. Approximately 27% of the members have received alert outreach from the CDE team. Patients may also interact with a Livongo CDE coach through a telephonic, one-to-one scheduled coaching session. Over 10% of members have completed at least one of these scheduled coaching sessions.

Other educational interactions include algorithmic, personalized messages that are sent through the meter in response to each BG reading. For example, if a BG value is below 50 mg/dL, the message on the BG meter will read “Your reading is very low, drink 4 oz of juice or take 4 glucose tabs and check BG again in 15 minutes.” If the BG value is above 400 mg/dL, the message will read “Drink a glass of water, take medication as prescribed and check BG again in 30 minutes.”

We hypothesized that the tools provided to Livongo members would decrease the likelihood of BG excursions outside of the normal glucose range (70-180 mg/dL or 3.9-10.0 mmol/L).

## Methods

### Study Design and Data

We collected registration data from individuals with diabetes who enrolled in the program between October 1, 2014 and December 30, 2015. This member-input data included sex, date of birth, diabetes type, insulin use, date of diagnoses, and other health-related information.

We collected BG data on each member using a cellular, cloud-connected BG meter that was shipped to each individual with test strips after registration. The BG readings and member-input details (such as how the member is feeling; relation of BG reading to meal or exercise, insulin taken, carbohydrates eaten) were automatically transmitted to the cloud in real time. All members used the same version of the glucose meter, and no significant device changes were made during the study period. Members with fewer than two total glucose readings were excluded from the analysis because no BG comparison could be made over time. No specific guidelines about BG testing frequency were given to members; rather, they were instructed to follow their health care provider’s advice.

### Key Variables

The main outcomes of interest were binary variables derived from the BG values collected from the meter. We defined a day with hyperglycemia as having at least one BG measure above 180 mg/dL in that day. We defined a day with hypoglycemia as having at least one BG measure below 70 mg/dL in that day.

We linked key variables from the registration data with the BG data using the individual identifier. These variables were age, sex (female, male), diabetes type (type 1, type 2, and unknown), and insulin use.

### Statistical Methods

We fit a series of logistic regression models to understand the predicted probability of hyperglycemia (days with BG >180 mg/dL) or hypoglycemia (days with BG <70 mg/dL) in months 2 to 12 compared with month 1.

In the absence of BG data prior to enrollment in Livongo, we used month 1 as a proxy for baseline. We hypothesized that members would have a lower probability of hyperglycemic and hypoglycemic events as their time in the program increased.

We included a fixed effect for each individual in order to control for all unobserved heterogeneity across members that may be correlated with the independent variables (ie, presence of a day with hypoglycemia or hyperglycemia in a given month), including lifestyle or the propensity to use technology that might influence BG control [11]. That is, *Y*_it_= *β*_oi_ + *β*_2_*month2* + *β*_3_*month3* +…+ *β*_12_*month12* + *ε*_it_, where *Y*_it_ is member-days with BG >180 or <70 mg/dL for individual *i* in month *t*; *β*_oi_ is the individual intercept that is swept out in the fixed-effects model; and *β*_s_… *β*_12_ represents the effects in months 2 to 12 compared with month 1.

Next, we stratified participants by insulin use, diabetes type, sex, insulin use, and age group (18-44, 54-64, ≥65). We conducted all modeling and statistical analyses using Stata 13 (StataCorp LP).

## Results

**Figure 1:**
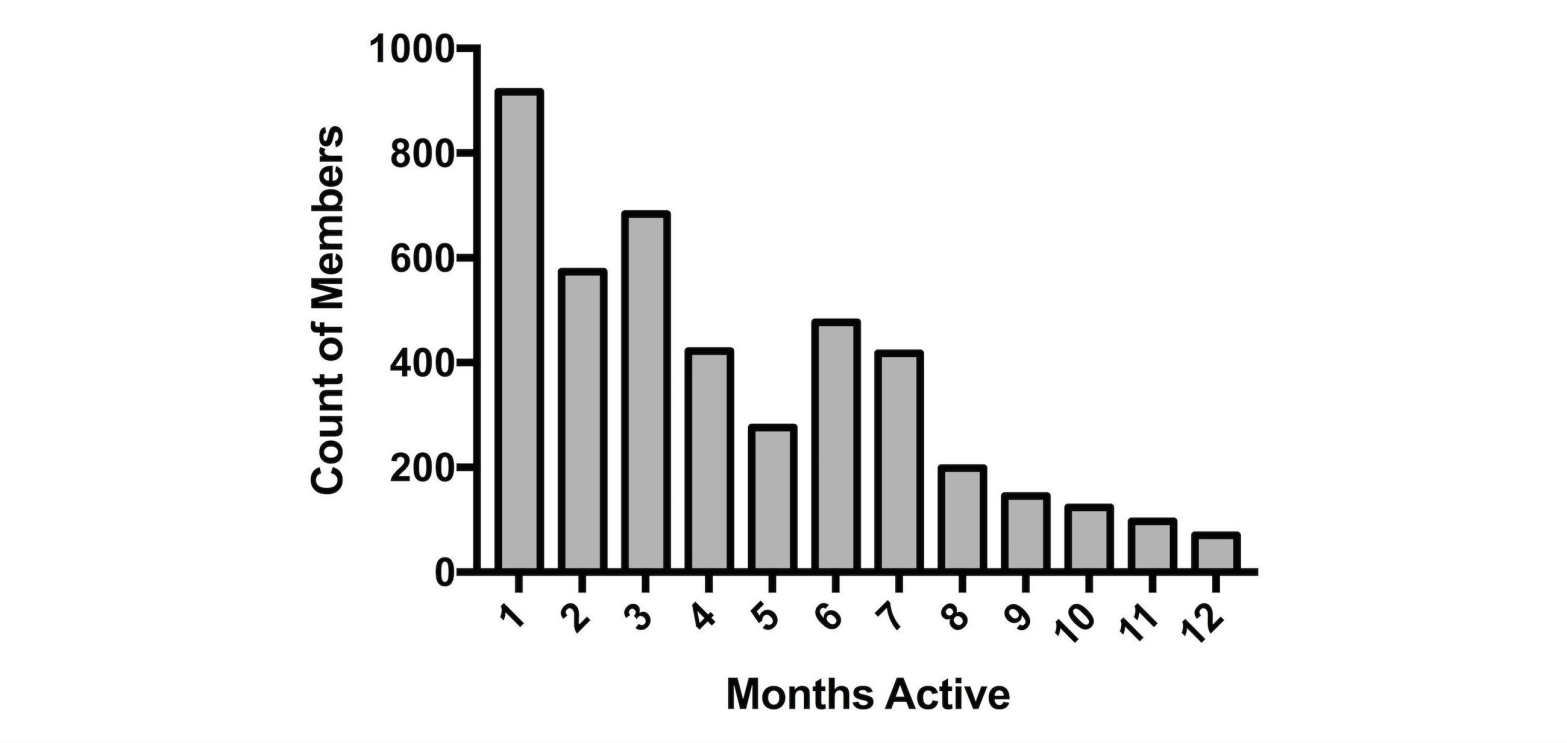
Distribution of members across months of Livongo program participation.

Of 4974 total Livongo members, 4544 had at least two BG measures during the period and were included in the analysis. Over the entire study period, members were enrolled for a mean of 95 days, and the mean total number of BG measurements was 114. Table 1 reports descriptive statistics for the sample population and Figure 1 shows the distribution of time in the program across members.

**Table 1:** Descriptive characteristics of the study population of Livongo members with more than 1 blood glucose (BG) check (n=4544)^a^.

Characteristics		n (%) or mean (SD)
Female, n, (%)		2499 (55.00)
**Age (years), n (%)**		
	18-44	1254 (27.60)
	45-64	2853 (62.79)
	≥65	436 (9.60)
**Diabetes type, n (%)**		
	1	709 (15.60)
	2	3303 (72.69)
	Unknown	532 (11.71)
**Insulin use, n (%)**		
	Yes	1704 (37.50)
	No	2213 (48.70)
No. days of Livongo participation, mean		95
Frequency of BG checks per day, mean		1.8
**Mean no. of days with BG >180 mg/dL, n (%)**		31.7 (33.4)
	Within-person SD	35.2%
	Between-person SD	34.2%
**Mean no. of days with BG <70 mg/dL, n (%)**		5.8 (6.1)
	Within-person SD	20.4%
	Between-person SD	11.9%
**BG (mg/dL), mean (SD)**		152 (54)
	Within-person SD	39
	Between-person SD	54

^a^Proportions do not always sum to 100 due to missing values.

**Figure 2:**
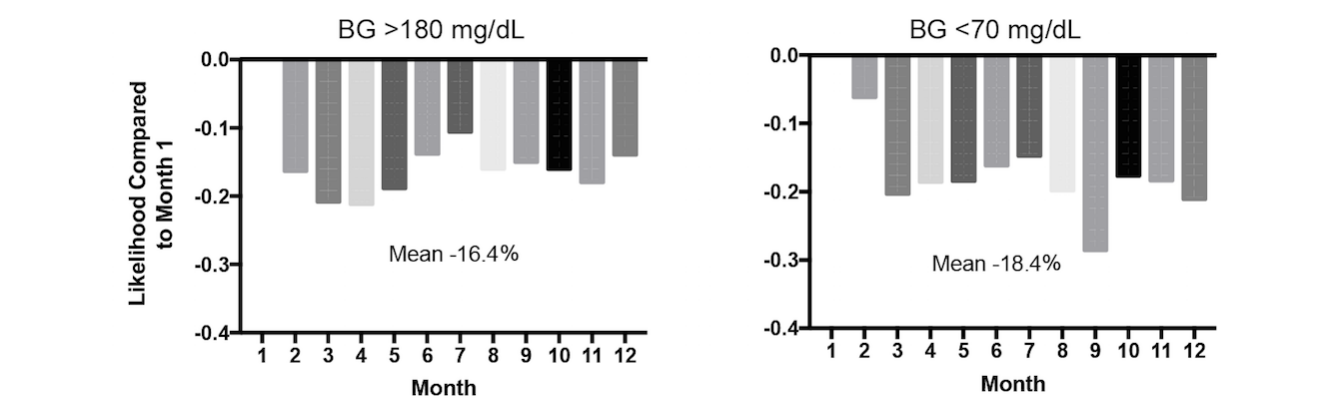
Likelihood of having blood glucose (BG) >180 mg/dL (left) and <70 mg/dL (right) in months 2-12, compared with month 1, for all members.

**Figure 3:**
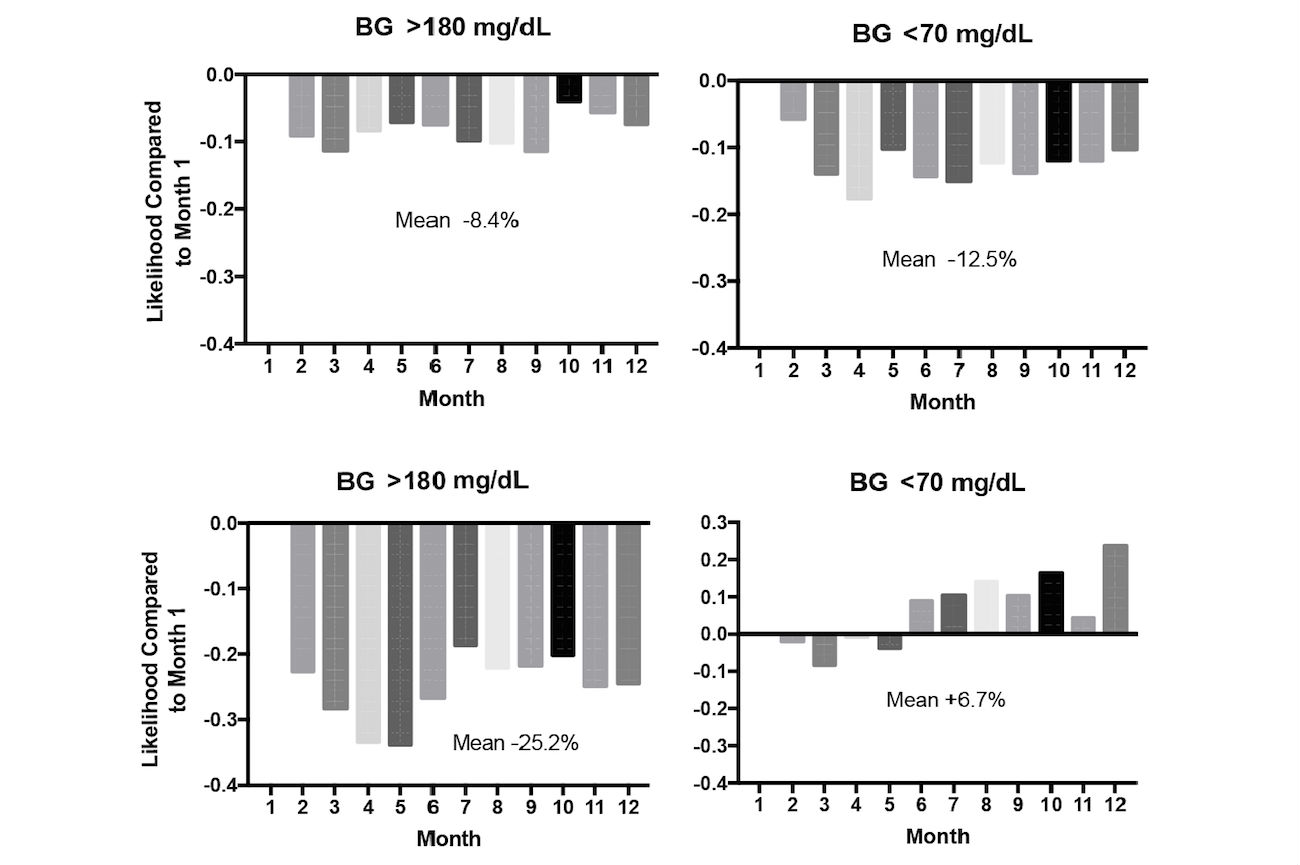
Likelihood of blood glucose (BG) >180 mg/dL (left, top and bottom) and <70 mg/dL (right, top and bottom) in months 2-12, compared with month 1, for users (top; n=1704) and nonusers (bottom; n=2213) of insulin.

Over the entire time period, the mean proportion of days with a BG value above 180 mg/dL was 33.4% with a within-person standard deviation of 35.2% and between-person standard deviation of 34.2%. The mean proportion of days with a BG value below 70 was 6.1% with a within-person standard deviation of 20.4% and a between-person standard deviation of 11.9%. More than half of individuals were female (55%), 15.6% had type 1 diabetes, 37.5% used insulin, and over half of the population (52.1%) had been active on Livongo for 4 or more months at the time of the analysis.

The mean BG for the entire population was 152 (SD 54) mg/dL (8.4, SD 3.0 mmol/L) over the 12 months and it was significantly higher for members with type 1 (163 mg/dL, or 9.0 mmol/L) compared to those with type 2 diabetes (150 mg/dL, or 8.3 mmol/L *P*<.001).

Figure 2 displays the predicted probabilities of hyperglycemia and hypoglycemia for the entire population. On average, the likelihood of having a day with a high BG reading (>180 mg/dL) in months 2-12 was 16.4% lower than in month 1. Specifically, in month 3, the likelihood of having a day with a hyperglycemic event was 21% lower than in month 1 (95% CI –0.195 to –0.131). For hypoglycemia, for each month, the likelihood of having a low BG reading (<70 mg/dL) was 18.4% lower than in month 1. In month 9, for example, the likelihood of having a hypoglycemic event was 29% lower (95% CI –0.105 to –0.018).

Figure 3 presents the results for hyperglycemia and hypoglycemia stratified by insulin use. For those who reported using insulin at Livongo program registration (n=1704), the likelihood of a day with hyperglycemia was decreased on average by 8.4% and the likelihood of hypoglycemia decreased by 12.5% in months 2-12 compared with month 1 (Figure 3, top). Individuals who reported not using insulin at registration experienced the biggest reduction in days with hyperglycemia by 25.2%, but also experienced an overall increase in hypoglycemic events of 6.7% starting in month 6 of the program (Figure 3, bottom). Changes in medication use were not systematically captured from individuals during the study period.

## Discussion

Our findings show that use of a technology-enabled connected glucose meter combined with open access to CDE support is effective in improving BG excursions for a diverse real-world population of adults with diabetes. Health care delivery based on episodic, in-person interactions between health care teams and patients does not fully address the real-time needs of patients with diabetes [12], which is a data-intensive condition requiring continuous management and the support of a multidisciplinary team. Tools provided by Livongo enable patients with diabetes to track their progress, and to collaborate with their providers and others such as family and friends who are positioned to provide critical support for patients who want to take an active role in managing their health. Additionally, this self-management program, like other technology-enabled solutions, can be a cost-effective strategy by empowering patients with engaging tools.

The limitations of this analysis include, first, that we did not have BG data for members prior to their enrollment in the Livongo program. We therefore used the first month of program participation as a proxy for baseline. This may underestimate the program’s impact, and our results should be interpreted as conservative.

Second, we were unable to capture medication or other treatment changes that can affect BG excursions, which could be relevant to the increased likelihood of hypoglycemia seen in members who reported not using insulin at the beginning of the program. In this group, 77.00% (3499/4544) of members reported taking oral medications and 16.99% (772/4544) reported taking no diabetes-related medications at baseline. Improved medication adherence and optimization of these members’ medication regimen to include starting insulin or an oral medication, such as a sulfonylurea, that can cause hypoglycemia could explain this finding.

Third, examination of overall diabetes control by laboratory-based hemoglobin A_1c_ assessment would have been useful to better understand whether the reduced frequency of hyperglycemic events in people with type 2 diabetes drove overall hemoglobin A_1c_ improvement, since the magnitude of either hypoglycemia or hyperglycemia was not factored into our model.

In summary, we examined a real-world population of people with diabetes using the connected Livongo meter and the Livongo for Diabetes Program to support diabetes self-care. Using the first month of the program as a baseline, the likelihood of both having a day with BG below 70 mg/dL and having a day with BG above 180 mg/dL decreased across the population. This is an important finding, as extreme hypoglycemic and hyperglycemic excursions are significant drivers of quality of life and health care costs for people with diabetes.

## Abbreviations

BG: blood glucose
CDE: certified diabetes educator
